# Immunobiological properties of selected natural and chemically modified phenylpropanoids

**DOI:** 10.2478/v10102-011-0002-1

**Published:** 2011-03

**Authors:** Juraj Harmatha, Zdeněk Zídek, Eva Kmoníčková, Jan Šmidrkal

**Affiliations:** 1 Institute of Organic Chemistry and Biochemistry, Academy of Sciences, v.v.i., Flemingovo nám. 2, CZ-166 10 Prague, Czech Republic; 2 Institute of Experimental Medicine, Academy of Sciences, v.v.i., Vídeňská 1083, CZ-142 20 Prague, Czech Republic; 3 Institute of Chemical Technology Prague, Faculty of Food and Biochemical Technology, Technická 3, CZ-16628 Prague, Czech Republic

**Keywords:** polyphenols, lignans, stilbenes, yatein, podophyllotoxin, resveratrol, inhibition of nitric oxide production, cytotoxicity assessment

## Abstract

Effects of natural and structurally transformed lignans compared with stilbenes or stilbenoids on production of nitric oxide (NO) triggered by lipopolysaccharide (LPS) and interferon-γ (IFN-γ), tested under *in vitro* conditions using murine resident peritoneal macrophages, are reviewed. Relation between the molecular structure and immunobiological activity was investigated, and implication of substituents, double bond stereochemistry, or cyclic attachments (double bond geometry fixation) was assessed. The focus was on lignans and stilbenoids because they were originally selected for a joint project of common interest to phytochemical and pharmacological investigation and because they represent well interesting and universally attractive groups of polyphenols with a feasible potential for therapeutic or nutraceutic utilization.

## Introduction

Phenolic compounds of plant origin integrate a large group of plant secondary metabolites with remarkably rich structural variations, and with a large variety of biological functions and activities. Functions are inherently involved in biochemical processes of the producing plant itself. Various activities take effect towards other organisms in symbiotic or defensive interactions. Plant phenolics are generally characterized as aromatic metabolites, possessing one or more hydroxyls attached directly to the aromatic (phenolic) moiety of the molecule, consequently with typical acidic properties. This property was, and still is, used for their detection, isolation or chemical transformation. It generates their chemical reactivity, and often also their biological activities. Plant phenolics appear to be in close biogenetic relations. Biosynthetic pathways of their formation differ often only in fractional details. Nevertheless, their structural variability can be surprisingly large. The most common types of plant phenolic compounds are usually classified according to the number of carbon atoms and their mutual correlation in the structure, as shown in Table 1 (modified according to Harborn, 1994; Harmatha, 2005).

**Table 1:** The most common plant phenolic compounds listed according to the count (content) of carbon atoms.

Composition	Count of carbons	Types of phenolic substances
C_6_	[6]	simple phenols, benzoquinones
C_6_-C_1_	[7]	phenolic acids / aldehydes
C_6_-C_2_	[8]	acetophenones, benzofurans
C_6_-C_3_	[9]	phenylpropanoids, benzopyranes (coumarins)
C_6_-C_4_	[10]	naphtoquinones
C_6_-C_5_	[11]	ageratochromenes (prekocens)
(C_6_)_2_	[12]	dibenzofurans, dibenzoquinones, biphenyls
C_6_-C_1_ -C_6_	[13]	dibenzopyranes, benzophenones, xanthones
C_6_-C_2_ -C_6_	[14]	**stilbenes,** antraquinones, phenanthrenes
C_6_-C_3_ -C_6_	[15]	flavonoids, isoflavones, chalcones, aurones
C_6_-C_4_ -C_6_	[16]	norlignans (diphenylbutadienes)
C_6_-C_5_ -C_6_	[17]	norlignans (conioids)
(C_6_-C_3_ )_2_	[18]	**lignans,** neolignans
(C_6_-C_3_ -C_6_)_2_	[30]	biflavonoids
(C_6_-C_3_ -C_6_)_n_	[n]	condensed tannins (flavolans)
(C_6_-C_3_ )_n_	[n]	lignins
(C_6_ )_n_	[n]	catecholmelanines

The basic structural differences, as generalized in Table 1, often show additional rich structural variability by incorporating different substituents. In the individual structures numerous phenolic hydroxyls are mostly present, forming polyhydroxyphenyl species, usually called polyphenols, which should never be confused with polymeric phenols.

## Phenylpropanoid

Phenylpropanoids are compounds containing the C_6_-C_3_ unit, or combined C_6_-C_3_-C_6_ unit, and their dimers or oligomers, as shown in Table 1, contain [9, 15, 18, 30 and n] carbon atoms. They clearly demonstrate a significant abundance of structures among just one type of phenolic secondary metabolites. Phenylpropanoids, as well as most of other types indicated in Table 1, are generated only by a limited number of basic biogenetic pathways, producing but a restricted number of two or three key intermediates. From these intermediates further hundreds up to thousands of so called periphery derivatives are formed usually by very simple but often specific enzymatic transformations. From the biogenetic point of view, phenylpropanoids are formed by the shikimate pathway (Umezawa, 2003). Our selection was focused on lignans, flavonoids and their closely related stilbenoids, and stilbene moiety containing phenylcoumarins.

## Lignans and their immunomodulatory activity

Lignans represent a characteristic and important group of biologically active polyphenolic metabolites, derived from two phenylpropanoide units (Umezawa, 2003; Harmatha & Dinan, 2003). Their rich and varied structural diversity and miscellaneous biological activities have always attracted considerable attention of phytochemists, occasionally including also botanists, pharmacologists, environmentalists and recently even experts for a superior and safe food production. At present, the growing interest in bioactive lignans, including also other polyphenols, is motivated by a potential use of these compounds as phytopharmaceuticals or nutraceuticals, *i.e.* biologically active food supplements, preventively efficient to protect health against the increasing number of chronic diseases, or health problems resulting from excessive environmental stress and working activity. The current wave of interest is indeed directed to the above mentioned aspects. However, no more than two decades ago it was still focused on chemical ecology, mainly on protection of plants against harmful organisms and adverse environmental effects. Lignans, along with other secondary metabolites, served also as important chemical characteristics (signs) for chemosystematic botanical classification (chemotaxonomy) in a time when genomics and proteomics had not yet been sufficiently developed and available for such purposes.

From the chemical point of view, lignans are formed by oxidative dimerization of two phenylpropanoid units and are defined by the link in the C_3_ side-chains between their two central carbon atoms (Harmatha, 2005). Thus they display a limited number of structural types (Harmatha & Dinan, 2003). Structural variability is, however, extended by the additional occurrence of one or two double bonds in the aliphatic part and of various substituents (mostly hydroxy, methoxy, methylenedioxy or glycosyl groups) on one or both aromatic rings and/or aliphatic carbon atoms. Even greater variability is associated with the inherent stereochemical disposition at the several chiral centers of the molecule (Harmatha, 2002, 2005).

**Figure 1:**
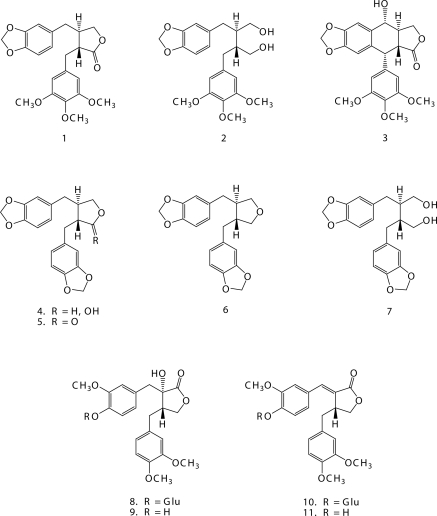
Chemical structures of lignans tested for immunomodulatory activity.

**Figure 2:**
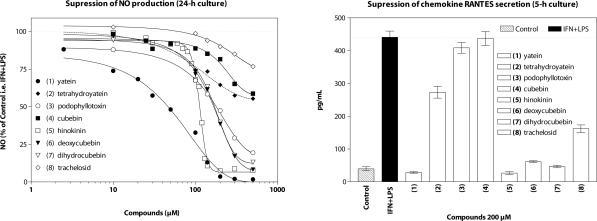
Immunobiological responses triggered by lipopolysacharide and interferon-γ under *in vitro* conditions using murine resident peritoneal macrophages. Reproduced from a preliminary report (Harmatha *et al*., 2004).

Our initial decision to investigate lignans resulted from our interest to use their characteristic and rich structural variability, and also their close biogenetic relation with other phenylpropanoids, for solving certain problems in the taxonomy of the order Cupresales (Erdtman & Harmatha, 1979). Later on, our interest turned more towards their insect feeding regulatory activity (Nawrot & Harmatha, 1994, Harmatha & Nawrot, 2002) and insect antihormonal effect, especially their assumed antagonistic influence reflecting their binding affinity to the ligand-binding site of the ecdysteroid receptor (Harmatha & Dinan, 2003). Recently we have attempted to learn more on the immuno-modulatory properties of lignans assessed in our well-established bioassay (Zídek *et al*., 2003). Effects of dibenzylbutane type lignans (Figure 1) on immunobiological responses triggered by lipopolysaccharide and interferon-γ were tested under *in vitro* conditions using murine resident peritoneal macrophages. Particularly, production of nitric oxide and secretion of cytokines and chemokines were investigated (Figure 2). The series of test agents encompassed the following lignan lactones: yatein (**1**)**,** podophyllotoxin (**3**), hinokinin (**5**) and tracheloside (**8**), one hemiacetal: cubebin (**4**), one ether, a reduced cubebin derivative: deoxycubebin (**6**) and two alcohols: tetrahydroyatein (**2**)**,** and dihydrocubebin (**7**). These dibenzylbutyne types of lignans were compared with the structurally related and well known biologically active (Ayres & Loike, 1990) aryltetraline type lactone podophyllotoxin (**3**). None of the test compounds posseses free phenolic hydroxyls, they contain mostly methoxy- and/or methylenedioxy- substituents.

Relation between the molecular structure and immunobiological activity was investigated, and implication of the functional groups was assessed.

The majority of the compounds tested represent natural substances. Yatein (**1**) was isolated from the heartwood of *Libocedrus yateensis* (Erdtman & Harmatha, 1979), (–)-cubebin (**4**) from seeds of *Piper cubeba* (Harmatha & Nawrot, 2002) and lignan glucoside tracheloside (**8**) from the medicinal plant *Leuzea carthamoides* (Harmatha *et al*., 2007). Hinokinin (**5**) and the structural analogues **2, 6** and **7** were prepared from yatein or cubebin by simple chemical transformations described earlier (Harmatha *et al*., 1982, Harmatha & Nawrot, 2002). Podophyllotoxin (**3**) was obtained from commercially available sources.

Natural lignan lactones possessing methoxy and/or methylenedioxy substituents showed a considerably high activity with a potency to be effective in assumed therapeutic exploitation. The highest activity was recorded for lignan lactones and diols. The maximum activity was achieved by the lignan lactone hinokinin (**5**), revealing a specific course of action. Selected data are shown in Figure 2. Recently were isolated, identified and tested additional lignans from *L. cartamoides*. One, glycosidic lignan, carthamoside (**10**) structurally related to tracheloside (**8**), differing only by the presence of a double bond in the aliphatic part (Harmatha *et al*., 2007) has showed a similar course of low activity as tracheloside (Figure 2). The two aglycones: trachelogenin (**9**) and carthamogenin (**11**), identical with the known 7,8-didehydroarctigenin, displayed activities comparable with the other lignans tested.

The majority of lignans are cytotoxic (Harmatha & Dinan, 2003), which is in accord with their well-known pharmacological activities (Slanina, 2000) especially as cancerostatic agents (Aires & Loike, 1990). It is known that the activity of lignans depends to a great extent on the type and position of substituents. At present, the dibenzylbutane type lignans with hydroxyls in the *meta-*positions of the aromatic rings, characteristic for natural enterolactones (mammalian gastrointestinal bacterial metabolites) (Raffaelli *et al*., 2002), are of particular interest for testing. These lignans share similarities with several estrogen-like substances (phytoestrogens) and are present in many food sources, even in wines (Nurmi *et al*., 2003). The observed similarity between enterolignans, phytoestrogens and immunoactive agents has led to the idea that they may play an important role as dietary supplements or active functional food components, nutraceuticals.

## Stilbenoids with immunoactive properties

Resveratrol and its structural analogues belong to a small but important group of plant secondary metabolites with a variously substituted stilbene unit. They possess an extensive range of biological activities (Fremont, 2000). Such compounds are widely abundant in nature (Šmidrkal *et al*., 2001), but can also be prepared by various synthetic approaches (Šmidrkal *et al*., 2001, Šmidrkal *et al*., 2010). Originally, we were attracted by their assumed antagonistic influence reflecting their binding affinity to the ligand-binding site of the insect ecdysteroid receptor (Harmatha & Dinan, 2003). At present, we have focused our attention on their possible immunobiological activity in mammalian cells, especially by comparing them with biogenetically related flavonoids and structurally related phenylcoumarins (Harmatha, 2002).

The testing set of stilbenes include *trans*-resveratrol (**1**), methyl-resveratrol (**2**), pinosylvin (**3**), and pterostilbene (**4**), as well as their homologues: trimethoxy-stilbenecarboxylic acid 7, its dihydro-derivative **8**, and their lactones: 7-hydroxy-3-(4-hydroxyphenyl)coumarin (**9**) and its dihydro-derivative **10**. These compounds were prepared by chemical synthesis using an original approach (Šmidrkal *et al*., 2010). Their structural differences are obvious, varying only by different type and position of substituents in compounds **1–8**, or by additional internal bonding, as in lactones **9, 10**. In all cases the stilbene (or dihydrostilbene in **8** and **10**) moiety is preserved. In order to compare the influence of stereochemical differences, *cis*-resveratrol (**5**) and isosteric 3,5,7-trihydroxyfenantren (**6**) were prepared by photo-transformation in a standard photo-reactor (Harmatha *et al*., 2002).

**Figure 3:**
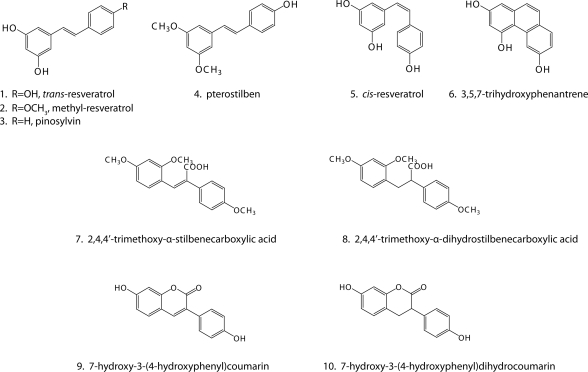
Chemical structures of stilbenes and related stilbenoids tested for immunomodulatory effects.

**Figure 4:**
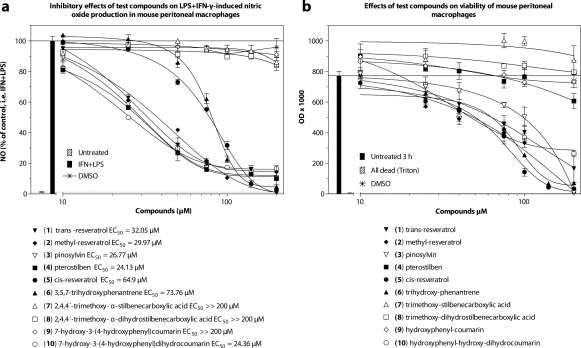
Inhibitory effect of stilbenes and related stilbenoids on LPS+IFN-γ induced NO production in mouse peritoneal macrophages (**a**); and effects of test compounds on viability of macrophages, i.e toxicity of test compounds (**b**). Reproduced from a preliminary report (Harmatha *et al*., 2008).

Our interest was focused on *trans*-resveratrol (**1**), pinosylvin (**3**), and pterostilbene (**4**), which usually occur as phytoalexins after bacterial infections (or during other environmental stress conditions) in their source organisms, *e.g.* grapevines (*Vitis vinifera*), pines (*Pinus sylvestris*), and various herbs, frequently used as foodstuffs. For a structure-activity relationship study, we prepared a series of structurally transformed analogues, such as **2, 5–10.** The trimethoxy-stilbenecarboxylic acids **7** and **8** did not inhibit NO production (Figure 4a), and from their lactones **9** and **10** only the dihydro derivative **10** displayed an activity comparable to the basic *trans*-stilbenes **1–4**. However, *cis*-resveratrol (**5**) and its isosteric 3,5,7-trihydroxyfenantren (**6**), conserving the same *cis* (*Z*) oriented stereochemistry, displayed a significant NO-inhibitory effect at concentrations higher than 50µM. Inhibition of NO was associated with the cytotoxicity of the compounds (Figure 4b). Interestingly, pterostilben (**4**) inhibited NO without affecting the viability of cells.

**Nitric oxide** (**NO**) **bioassay**. Isolation and cultivation of murine peritoneal macrophages for bioassay was performed in a routine way (Zídek *et al*., 2003). Macrophages were cultured 24 hrs in the presence of test compounds, applied in the presence of NO-priming immune stimuli, *i.e.* murine recombinant interferon-γ (IFN-γ) and lipopolysaccharide (LPS). The concentration of nitrites in supernatants of cells was detected by Griess reagent. The absorbance at 540 nm was recorded using a micro-plate spectrophotometer.

**Cytotoxicity assay**. Viability of cells was determined using a colorimetric assay based on cleavage of the tetrazolium salt WST-1 by mitochondrial dehydrogenases in viable cells (Roche Diagnostics, Mannheim, Germany). The cells were cultured (1×10^6^/mL) as described above. After the 24-h culture, WST-1 was added and the cells were kept in the Heraeus incubator at 37°C for additional 3 h. Optical density at 450–690 nm was evaluated. The test compounds were dissolved in dimethylsulfoxide (DMSO). The final concentration of DMSO was lower than 0.02%. This amount was devoid of any effects on NO production and on cell viability.

## Conclusion

Stilbenoid immunomodulatory activities can be compared with activities of biogenetically related lignans, exhibiting similar effects in the same immuno-assays. Thus our results extended the field of bioactivity of both lignans and stilbenoids, including the opportunity of their potential therapeutic or nutraceutic utilization.

However, for further advanced investigation concerning oxidative stress mediated inflammation, related to autoimmune diseases (*e.g.* rheumatoid arthritis), after preliminary screening tests most stilbenes were selected (Perečko *et al*., 2008; Perečko *et al*., 2010; Jančinová *et al*., 2010; Mačičková *et al*., 2010), along with some of their structural analogues, *e.g.* phenylcoumarins (Drábiková *et al*., 2010). Lignans failed to exhibit the same application potential in the above mentioned stress induced diseases.
